# Ulvan and *Ulva* oligosaccharides: a systematic review of structure, preparation, biological activities and applications

**DOI:** 10.1186/s40643-023-00690-z

**Published:** 2023-09-25

**Authors:** Chen Li, Tiancheng Tang, Yuguang Du, Ling Jiang, Zhong Yao, Limin Ning, Benwei Zhu

**Affiliations:** 1 School of Medicine and Holistic Integrated Medicine, Nanjing University of Chinese Medicine, Nanjing, 210023 China; 2 College of Food Science and Light Industry, Nanjing Tech University, Nanjing, 211816 Jiangsu China; 3 State Key Laboratory of Biochemical Engineering, Institute of Process Engineering, Chinese Academy of Sciences, Beijing, China

**Keywords:** Structure, Activity, Applications, Ulvan, *Ulva* oligosaccharide

## Abstract

*Ulva* is one of the main green algae causing green tide disasters. Ulvan is the primarily component polysaccharide of the cell wall of *Ulva* and its complex structure and monosaccharide composition resulted in various biological activities. However, the high-value and effective utilization of extracted ulvan have been obstructed by limitations ranging from large molecular weight and low solubility to poor bioavailability. *Ulva* oligosaccharide obtained by degrading ulvan can not only ideally retain the various biological activities of ulvan very well but also effectively solve the problems of low solubility and poor bioavailability. The preparation and biological activity studies of ulvan and *Ulva* oligosaccharides have become a hot spot in the field of marine biological resources development research. At present, the comprehensive reviews of ulvan and *Ulva* oligosaccharides are still scarce. What are overviewed in this paper are the chemical composition, structure, extraction, and purification of ulvan and *Ulva* oligosaccharides, where research progress on the biological activities of ulvan and *Ulva* oligosaccharides is summarized and prospected. A theoretical and practical basis has been provided for further research on ulvan and *Ulva* oligosaccharides, as well as the high-value development and effective utilization of marine algae resources.

**Graphical Abstract:**

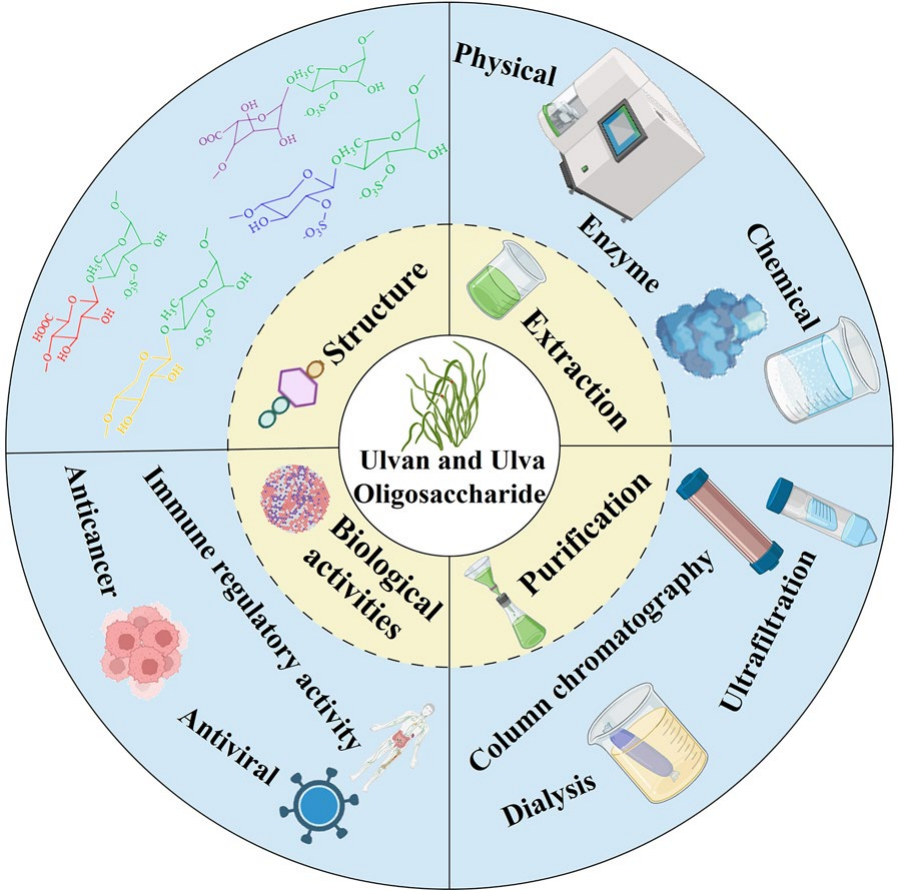

## Introduction

Since 2008, ecological disasters such as green tides have frequently occurred in the Yellow Sea in China, and *Ulva* is one of the main algae that form green tides (Chen et al. 2022b; Wang et al. 2015; Ye et al. 2011; Zheng et al. 2022). *Ulva* (*Ulva* sp.) belongs to the *Ulva* family in the *Ulva* genus of Chlorophyta, and the common types of *Ulva* include *U. prolifera*, *U. pertusa*, and *U. compressa* (Beer 2023; Van Alstyne et al. 2015; Wang et al. 2015). *Ulva* has a high nutritional value, such as sulfated polysaccharides, lipids, proteins, dietary fibers, and vitamins, which support their use in medical applications (Cadar et al. 2023; Kidgell et al. 2019). Therefore, it has a wide range of application prospects in health food, agricultural feed, and fish–shrimp farming (Kidgell et al. 2019). Ulvan is the main water-soluble sulfated polysaccharide existing in the cell wall of *Ulva* algae (Ning et al. 2022; Zheng et al. 2022). It was found that ulvan was mainly composed of rhamnose, iduronic acid, xylose, glucuronic acid, and a small amount of galactose, and the composition of the monosaccharide and the degree of sulfation varied with the species, harvest season, cultivation methods, growth environment, and extraction methods of *Ulva* sp. (Kidgell et al. 2019; Ning et al. 2022; Olasehinde et al. 2019; Olsson et al. 2020). Ulvan, as the main active component of *Ulva* sp., has a variety of biological activities, such as anti-inflammatory (Kidgell et al. 2020), anticoagulant (Faggio et al. 2016), anti-tumor (Pradhan et al. 2020), antioxidant (Pradhan et al. 2022), anti-hyperlipidemia (Yuan et al. 2018), immune regulation (Fernández-Díaz et al. 2017), and plant growth-promoting activities (Shefer et al. 2022). Therefore, ulvan has application prospects in biomedicine, cosmetics, food, health products, and other industries (Berri et al. 2017; Cindana Mo’o et al. 2020; Morelli et al. 2019; Ning et al. 2022; Samah and Hadear 2019; Yaich et al. 2017). However, the high-value development and utilization of ulvan resources have been limited by their large molecular weight, poor solubility, and low bioavailability. On the contrary, the *Ulva* oligosaccharide obtained from the degradation of ulvan not only retains the biological activities of the original polysaccharide well but also improves the defects of the insolubility of ulvan in water and the low bioavailability, which makes the preparation of *Ulva* oligosaccharide and the research on its biological activity a hot spot in the field of the development and research of marine biological resources (Ning et al. 2022; Paulert et al. 2021).

At present, the research reports related to ulvan and its oligosaccharide are increasing, but there are no relevant papers to summarize and discuss the current research progress. In this paper, the chemical composition, structure, extraction, purification, and biological activities research reports of ulvan are systematically summarized, and the preparation methods of *Ulva* oligosaccharides and the activities of *Ulva* oligosaccharides are reviewed and analyzed. At the end of the paper, the main problems as well as technical bottlenecks, and application prospects in various fields in the development and utilization of ulvan are analyzed and prospected, providing a theoretical basis and research basis for promoting the effective utilization of ulvan, a kind of marine green biomass resource.

## Chemical composition of ulvan

Ulvan is structurally more complex than other algal polysaccharides due to the complexity of their monosaccharide composition, glycosidic bonding, and group modifications. The chemical composition of ulvan will be affected by the type of *Ulva* sp. as the extraction source, the harvest season, and the extraction and purification methods of ulvan, so its composition is more complex (Table 1). Kidgella et al. analyzed the monosaccharide composition of the ulvan from blade (*U. australis*, *U. rigida*, *U*. sp. B, and *Ulva* sp.) and filamentous (*U. flexuosa*, *U. compressa*, *U. prolifera*, and *U. ralfsii*) species of cultivated *Ulva* and found that there was a large difference in the monosaccharide composition of them (Kidgell et al. 2021). Among them, the ulvan from blade species of cultivated *Ulva* mainly consists of rhamnose (~ 49 mol%), followed by glucuronic acid (~ 23.83 mol%), and the proportion of xylose and iduronic acid varies with the different types of *Ulva* sp. Ulvan from filamentous species of cultivated *Ulva* are quite different. The content of rhamnose in the ulvan from *U. prolifera* and *U. flexuosa* can reach 56 mol% and 60 mol%. Conversely, the content of rhamnose in the ulvan from *U. ralfsii* and *U. compressa* is about 43 mol%. However, the proportion of iduronic acid in ulvan from filamentous species of cultivated *Ulva* (~ 7 mol%) is generally lower than that from blade species of cultivated *Ulva* (~ 14 mol%). In addition, the content of iduronic acid in ulvan from *U. rigida* is the highest, reaching 18 mol%, and the proportion of galactose in ulvan from *U. ralfsii* reached 16 mol%, which was far more than that of ulvan from other *Ulva* sp. Samarasinghea et al. analyzed the monosaccharide composition of *Ulva* harvested at different months and found that the main composition of ulvan was not different, but the content of different types of monosaccharides was quite different (Samarasinghe et al. 2021). For example, the dry matter contents of rhamnose, xylose, galactose, glucose, and uronic acid in ulvan harvested in June were 3.65, 0.43, 0.41, 0.32, and 0.62 g/100 g, respectively; the dry matter contents of rhamnose, xylose, galactose, glucose, and uronic acid in ulvan collected in August were 0.84, 0.33, 0.22, 0.75, and 1.92 g/100 g, respectively. It can be clearly seen that the ulvan harvested in August has a higher content of glucose and uronic acid than the ulvan harvested in June. Furthermore, when Olsson et al. studied the effect of cultivation conditions (such as temperature, irradiance, pCO_2_, nitrogen, and phosphate) on the monosaccharide composition of ulvan, and they found that low sulfate concentration and high temperature could promote the increase of monosaccharide content, while increasing irradiance and temperature could increase the concentration of rhamnose and iduronic acid in the ulvan(Olsson et al. 2020). Guidara et al. extracted ulvan from *Ulva lactuca* by acid extraction (CA) and enzymatic chemical extraction (EE), respectively, and analyzed their monosaccharide components (Guidara et al. 2020). The results showed that the content of rhamnose and xylose in ulvan CA1 obtained by CA was higher than that of ulvan EE extracted by EE, and the content of uronic acid and glucose in EE was higher than that in CA1. The above research results show that the chemical composition of ulvan varies with *Ulva* sp., growth environment, harvest time, and extraction method, and this phenomenon is also common in the composition analysis of other algal polysaccharides (Benslima et al. 2021).

**Table 1:** The summary of the chemical composition for each *Ulva* sp

Sources	Monosaccharide (mol%)
*U. australis*	Rhamnose(51) Glucuronic acid(18) Xylose(22) Iduronic acid(7)
*U. rigida*	Rhamnose(49) Glucuronic acid(26) Xylose(6) Iduronic acid(18)
*U*. sp. B	Rhamnose(48) Glucuronic acid(31) Xylose(7) Iduronic acid(11)
*Ulva* sp.	Rhamnose(47) Glucuronic acid(20) Xylose(19) Iduronic acid(10)
*U. flexuosa*	Rhamnose(56) Glucuronic acid(21) Xylose(15) Iduronic acid(6)
*U. compressa*	Rhamnose(47) Glucuronic acid(24) Xylose(17) Iduronic acid(7)
*U. prolifera*	Rhamnose(60) Glucuronic acid(17) Xylose(15) Iduronic acid(7)
*U. ralfsii* (cult.)	Rhamnose(38) Glucuronic acid(24) Xylose(16) Iduronic acid(4) Galactose(16)
*U. ralfsii* (wild)	Rhamnose(43) Glucuronic acid(26) Xylose(14) Iduronic acid(6) Galactose(10)

## The structure of ulvan and *Ulva* oligosaccharide

**Fig. 1:**
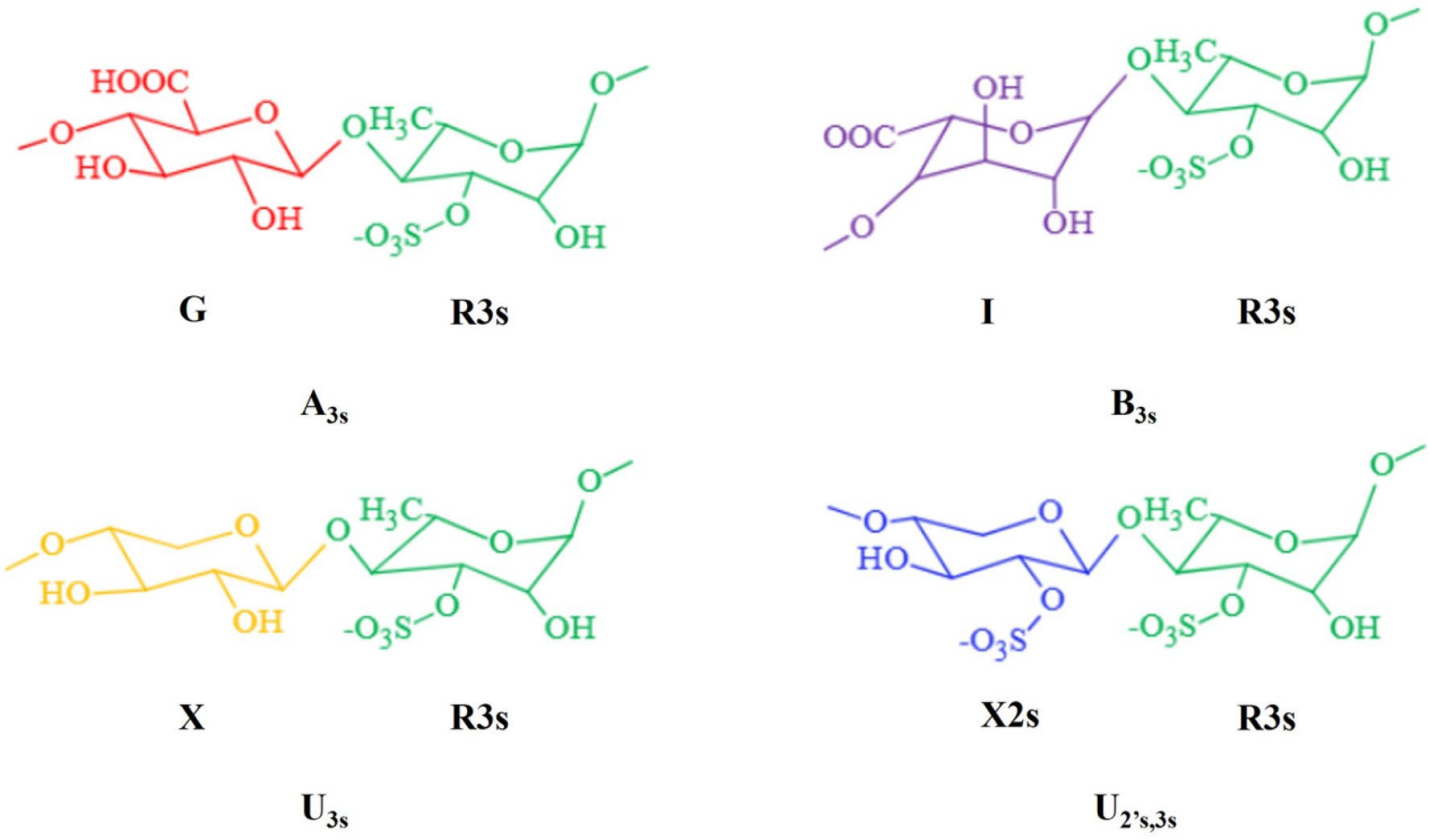
The structures of main disaccharide units in ulvan

The complex monosaccharide composition, different connection modes between monosaccharides, the existence of complex and diverse group modifications, and branched structures make the structure of ulvan far more complex than that of other algal polysaccharides such as alginate, carrageenan, and agarose (Stender et al. 2019). Therefore, it is difficult to elucidate the structure of ulvan. Lahaye et al. studied the structure of ulvan from *U. rigida* (Lahaye and Robic 2007). Six sample structures from the Canary Islands, namely, Δ(1 → 4)*α*-l-Rha3S(1 → 4)*β*-d-Xyl2S(1 → 4)l-Rha3S, Δ(1 → 4)*α*-l-Rha3S(1 → 4)*β*-d-Xyl(1 → 4)*α*-l-Rha3S(1 → 4)*β*-d-Xyl(1 → 4)l-Rha3S, Δ(1 → 4)*α*-l-Rha3S(1 → 4)*β*-d-Xyl2S(1 → 4)*α*-l-Rha3S(1 → 4)*β*-d-Xyl(1 → 4)l-Rha3S, Δ (1 → 4)*α*-l-Rha3S (1 → 4) *β*-d-Xyl2S(1 → 4) *α-*l-Rha3S(1 → 4)*β*-d-Xyl(1 → 4)*α*-l-Rha3S(1 → 4)*β*-d-Xyl(1 → 4)l-Rha3S, Δ(1 → 4)l-Rha3S, and Δ(1 → 4)*α*-l-Rha3S(1 → 4)*β*-d-Xyl(1 → 4)l-Rha3S, were determined. And four structures of sample from the Brittany, Δ(1 → 4)*α*-l-Rha3S(1 → 4)*β*-d-GlcA(1 → 4)l-Rha3S, Δ(1 → 4)*α*-l-Rha3S(1 → 4)*β*-d-Xyl2S(1 → 4)l-Rha3S, Δ(1 → 4)[*β*-d-GlcA(1 → 2)]*α*-l-Rha3S(1 → 4)*β*-d-Xyl(1 → 4)l-Rha3S(20), and Δ(1 → 4)[*β*-d-GlcA(1 → 2)]*α*-l-Rha3S(1 → 4)*β*-d-Xyl2S(1 → 4)l-Rha3S, were also found, along with the same oligosaccharide structure as the previous sample, Δ(1 → 4)l-Rha3S and Δ(1 → 4)*α*-l-Rha3S(1 → 4)*β*-d-Xyl(1 → 4)l-Rha3S, where Δ refers to the unsaturated uronic acid 4-deoxy-l-threo-hex-4-enopyranosiduronic acid at the non-reducing end. The main disaccharide repeat structures of ulvan are A_3s_ [→ 4)*β*-d-GlcA(1 → 4)-*α*-l-Rha3S(1 →] and B_3s_ [→ 4)*α*-l-IdoA(1 → 4)-*α*-l-Rha3S(1 →] from different *Ulva* sp. samples. In addition, the analysis of these structures shows that there are another two repeating disaccharide units, U_3s_ [→ 4)*β*-d-Xyl(1 → 4)-*α*-l-Rha3S(1 →] and U_2’s,3s_ [→ 4)*β*-d-Xyl2S(1 → 4)-*α*-l-Rha3S(1 →]. Thanh et al. extracted highly purified ulvan and analyzed its structure by IR, NMR, SEC-MALL, and ESI–MS(Thanh et al. 2016). The study found that there were repeat disaccharide units of A_3s_ [→ 4)-*β*-d-GlcA-(1 → 4) -*α*-l-Rha3S-(1 →], GlcA-(1 → 2)-Xyl and GlcA-(1 → 2)-Rha in the ulvan extracted from *Ulva* sp., of which A_3s_ [→ 4)-*β*-d-GlcA-(1 → 4)-*α*-l-Rha3S-(1 →] was the main disaccharide repeat unit. Chi et al. used ulvan lyase to cleave ulvan from *U. clathrata* through *β* elimination reaction and obtained three degradation products with different molecular weights, UO-1, UO-2, and UO-3, and carried out structural analysis on them, respectively (Chi et al. 2020a). UO-1 and UO-2 have lower molecular weights and are disaccharides, _D_-ΔGlcA-(1 → 4)-*α*/*β*-_L_-Rha3S, and tetrasaccharides, _D_-ΔGlcA-(1 → 4)-*α*-l-Rha3S-(1 → 4)-*β*-d-Xyl-(1 → 4)-*α*/*β*-l-Rha3S, respectively. The study of the UO-3 structure with a large molecular weight found that it was mainly composed of A_3s_ type and U_3s_ type disaccharide repeat units, and there were also U_2's, 3s_ type disaccharide repeat units. It can be seen that ulvan is a complex polysaccharide mainly composed of A_3s_ or B_3s_ type disaccharide repeat units and a small number of U_3s_ or U_2's, 3s_ type disaccharide repeat units (Fig. 1), and the content of different disaccharide repeat units will be affected by different sources of *Ulva* sp. Therefore, the research on the structure of ulvan will help strengthen the high-value development and utilization of ulvan resources.

## Preparation of ulvan and its oligosaccharide

The preparation of ulvan can be divided into two steps: extraction and purification, and there are many reported methods for extracting and purifying ulvan. According to the extraction and purification process, we classify the extraction methods into the following three categories: solution extraction, physical-assisted extraction, and enzyme-assisted extraction. Similarly, the purification methods of ulvan can also be classified into three categories: dialysis, ultrafiltration, and column chromatography, of which column chromatography can be further divided into ion exchange chromatography and gel filtration chromatography.

### Extraction methods of ulvan

The extraction method of ulvan is very similar to that of other algae polysaccharides, mainly using solution extraction and two auxiliary extraction methods based on it, namely, physical-assisted and enzyme-assisted extraction. Source, extraction method, and yield of ulvan extracted from different *Ulva* sp. are shown in Table 2.

**Table 2:** The summary of extraction methods for ulvan

Extraction methods	Source	Extraction Time (min)	Temperature (^o^C)	Yield (%)	Refs.
Hot water extraction (Temperature below 100 °C)	*Cladophora glomerata*	420	75–85	16.23	(Pankiewicz et al. 2016)
	*U*. *pertusa Kjellm*	180	90	17.8 ± 0.6	(Chen et al. 2021)
	*U*. *lactuca*	180	90	11 ± 3	(Wahlström et al. 2020)
	*U*. *rigida*	60	90–100	7	(Paradossi et al. 1999)
	*U*. *clathrata*	120	100	11.27	(Chi et al. 2020a)
	*Ulva intestinalis*	90	100	15.2	(Klongklaew et al. 2021)
Pressurized hot water	*U. fasciata*	60	110	19.5	(Paulert et al. 2021)
	*U*. *Rigida*	30	130	24.3	(Toskas et al. 2011)
Acid extraction	*U*. *ohnoi*, *U*. *tepida*, *U*. *prolifera*	180	37	3.5, 3.9, 6.7	(Glasson et al. 2022)
	*U*. *rotundata*	60	85	20.3	(Robic et al. 2009)
	*U*. *ohnoi*	60	85	8.1 ± 1.0	(Glasson et al. 2017)
	*U*. *lactuca*	60	80	13.06	(Yaich et al. 2014)
	*U*. *lactuca*	240	90	18 ± 2	(Wahlström et al. 2020)
	*U*. *lactuca*	180	90	32.67	(Yaich et al. 2013)
	*U.* sp. (foliose), *U.* sp. (filamentous)	240	90	41 ± 2.2, 39 ± 1.9	(Manikandan and Lens 2022)
Weak alkali extraction	*U*. *ohnoi*	60	85	4.3 ± 0.5	(Glasson et al. 2017)
Enzyme-assisted extraction	*U*. *pertusa Kjellm*	180	50	25.3 ± 1.3	(Chen et al. 2021)
	*U*. *armoricana*	195	50	35.3 ± 0.3	(Hardouin et al. 2016)
Ultrasonic-assisted extraction	*U*. *pertusa Kjellm*	180	90	20.6 ± 1.2	(Chen et al. 2021)
Microwave-assisted extraction	*U*. *ohnoi*, *U*. *meridionalis*	60	85	36.5 ± 3.1,40.4 ± 3.2	(Tsubaki et al. 2016)
	*U. prolifera*	15	120	36.38 ± 0.94	(Yuan et al. 2018)
Enzymatic and chemical extraction	*U*. *lactuca*	300	50	17.14	(Yaich et al. 2014)
Ultrasound and enzyme-assisted extraction	*U*. *pertusa Kjellm*	180	50	26.7 ± 0.9	(Chen et al. 2021)

#### Solution extraction

Solution extraction can be further subdivided into hot water extraction, pressurized hot water extraction, acid extraction, and weak alkaline extraction. Hot water extraction is a traditional polysaccharide extraction method and using that method the solubility of polysaccharides in water will rise with the increasing temperature. And the ratio of material to liquid, the temperature of hot water, extraction time, the extraction times, and other factors will affect the yield of polysaccharide. Pankiewicz et al. stirred and extracted ulvan for 7 h in a hot water bath at 75–85 ℃ according to the ratio of material to liquid of 1:30, centrifuged and filtered the supernatant rich in polysaccharide, and concentrated it (Pankiewicz et al. 2016). The yield of polysaccharide was 16.23%. Wahlström et al. first mixed with 70% ethanol according to the feed-to-liquid ratio of 1:10, then extracted at room temperature at 300 rpm for 8 h, centrifuged to obtain the polysaccharide sediment, and washed it with ethanol three times (Wahlström et al. 2020). Then, according to the material-to-liquid ratio of 1:16, add it to the hot mixer of ultrapure water to extract for 3 h at 90 ℃ and 750 revolutions. Collect the supernatant and extract it twice at the material-to-liquid ratio of 1:14 and then freeze dry it. The yield of ulvan is 11 ± 3%. Chen et al. extracted ulvan for 3 h in a 90 ℃ water bath using a material-to-liquid ratio of 1:20. After centrifugation and concentration, 95% ethanol was used for alcohol precipitation, and then 95% ethanol was used for repeated washing three times and dried in a vacuum oven at 50 ℃ for 1 h to achieve constant weight (Chen et al. 2021). The yield was 17.8 ± 0.6%. Gaio et al*.* selected the ratio of material to liquid of 1:10 to extract for 1 h in hot water at 90–100 ℃, repeated once, recovered the supernatant, and precipitated it in 1 volume of mixed water/ethanol at a ratio of 1:1 (v/v), and the polysaccharide yield was 7% (Paradossi et al. 1999). Chi et al. selected a material-to-liquid ratio of 1:30 to extract for 2 h in hot water at 100 ℃, centrifuged the supernatant, concentrated it, dialyzed it in cellulose membranes, and then precipitated it with 95% ethanol (Chi et al. 2020a). The polysaccharide yield was 11.27%. Klongklaewad et al. boiled for 90 min in the autoclave according to the feed-to-liquid ratio of 1:100, filtered the solid residues to retain the supernatant, and freeze-dried, and the polysaccharide yield was 15.2% (Klongklaew et al. 2021). However, hot water extraction has disadvantages, including a low extraction yield and a long extraction time. Pressurized hot water extraction is regarded as a green and efficient technique to extract solid samples with liquid water (Plaza and Marina 2019). This method intensified the destruction of the cell structure of *Ulva* sp. under higher temperature and pressure and increased the content of dissolved polysaccharides, thus improving the yield. Paulertab et al. selected a material/liquid ratio of 1:40 to extract ulvan for 1 h in pressurized hot water at 110 ℃. The recovered water extract was concentrated and then precipitated with ethanol, with a yield of 19.5%. Similarly, Toskasa et al. used pressurized hot water with a material-to-liquid ratio of 1:20 at 130 ℃ to extract for 30 min, filter it, and recover the hot water solution. After concentrating the hot water solution, use 95% ethanol for alcohol precipitation, then use 95% ethanol for repeated washing three times, and then dry it at 80 ℃. The average yield can reach 24.3%. Compared with the traditional hot water extraction method, the high-pressure hot water extraction method has the characteristics of a short extraction time and a high yield. However, the exorbitant temperature will cause the self-degradation of ulvan, which will lead to a decrease in its yield (Podolean et al. 2022). Therefore, it is helpful to optimize the hot water extraction scheme of ulvan according to the needs of the follow-up study of ulvan.

On the basis of hot water extraction, the extraction process of ulvan can be improved by adjusting the pH value of the extraction solution. Yaich et al. used the acid extraction method with a material-to-liquid ratio of 1:16.7 to extract ulvan (Yaich et al. 2013). Through multifactorial and multi-level experiments, they optimized the three factors, pH value, extraction temperature, and extraction time, and obtained the best extraction process, which is a pH value of 2, an extraction temperature of 90 ℃, and an extraction time of 3 h. The yield of ulvan under this process is 32.67%. Wahlström et al. added 0.01 M HCl (pH 2.0) according to the feed-to-liquid ratio of 1:25, heated it to 90 ℃, and extracted it for 4 h (Wahlström et al. 2020). The supernatant was recovered by centrifugation, dialyzed for 48 h, and then ethanol precipitated the dialysate. The precipitated part was collected and freeze-dried. The yield of ulvan was 18 ± 2%. Glasson et al. added the pretreated *Ulva* sp. to 1L of 0.05 M HCl, heated it to 85 ℃, extracted it for 1 h, then vacuum filtered it, adjusted the pH to 7 with 1 M NaOH, concentrated it, and freeze-dried it (Glasson et al. 2017). The polysaccharide yield was 8.1 ± 1.0%. Christopher et al. added 0.05 mol/L HCl solution adjusted to pH 2 by 1 M NaOH into *U. Ohnoi*, *U. tepida,* and *U. prolifera* from three different sources at a ratio of 1:25 to extract for 3 h at 37 ℃, filtered and recovered the extract, then filtered the extract in vacuum, concentrated by ultrafiltration, and freeze-dried after dialysis (Glasson et al. 2022). The final yield of ulvan was 3.5%, 3.9%, and 6.7%, respectively. In addition to HCl, Manikandan et al. also tried to use citric acid, a green chemical, to extract ulvan from foliose *Ulva* sp. and filamentous *Ulva* sp. and optimized it (Manikandan and Lens 2022). The polysaccharide extraction rates of foliose *Ulva* sp. and filamentous *Ulva* sp. were 0.41 (± 0.022) g/g and 0.39 (± 0.019) g/g, respectively, When the extraction temperature was 90 °C, the extraction time was 4 h, the minimum *Ulva* sp. load was 3 wt%, and the citric acid concentration was 1 wt%. Conversely, Christopher et al. tried to extract ulvan with sodium oxalate, but the yield was only 4.3 ± 0.5% and the content of protein was higher than that extracted by HCl (Glasson et al. 2017). Compared with hot water extraction, chemical extraction also improves the yield of ulvan by increasing the temperature and prolonging the extraction time, but its extraction conditions are more stringent. This is not only bad for industrial production but will also greatly increase the environmental maintenance costs in the production process.

#### Physical-assisted extraction

The physical-assisted extraction method mainly uses physical methods other than heating to help destroy the cell wall structure of *Ulva* sp., making it easier to extract polysaccharides from *Ulva* sp., thereby shortening the extraction time and improving the yield of ulvan. Chen et al. used the ultrasonic-assisted method to extract ulvan, mixed it according to the material-to-liquid ratio of 1:20, first treated it with ultrasonic for 30 min, and then extracted it in a 90 ℃ water bath for 2.5 h, with an extraction rate of 20.6 ± 1.2% (Chen et al. 2021). However, Tsubaki et al. used microwave-assisted technology to extract ulvan at 100 ℃ to 180 ℃ after mixing according to the material-to-liquid ratio of 1:20 (Tsubaki et al. 2016). At 160 ℃, the extraction rates of ulvan reached 40.4 ± 3.2% (*U. meridionalis*) and 36.5 ± 3.1% (*U. ohnoi*). Yuan et al. mixed the mixture in a 0.01 M HCl solution according to the feed–liquid ratio of 1:20 and received microwave irradiation at 120 ℃ for 15 min (Yuan et al. 2018). The yield of ulvan was 36.38 ± 0.94%. The cell wall of *Ulva* sp. was greatly damaged by the method of physical-assisted extraction, which greatly improved the efficiency of the next hot water extraction. In particular, the yield of microwave-assisted extraction is about 2–3 times that of traditional hot water extraction.

#### Enzyme-assisted extraction

Enzyme-assisted extraction is a new method to improve the yield of polysaccharides based on hot water extraction and enzyme technology. Before reaction, add cellulase and pectinase that can degrade cellulose, hemicellulose, and pectin in the cell wall of *Ulva* sp., and release more ulvan by destroying the structure of the cell wall (Fernandes et al. 2019). Chen et al. prepared the solution according to the material-to-liquid ratio of 1:20 and added 1 M HCl to adjust the pH value to 4.5 (Chen et al. 2021). First, they reacted with cellulase and the mixture in a water bath at 50 ℃ for 2.5 h, then raised the temperature to 90 ℃, and inactivated the enzyme for 30 min. The yield of ulvan was 25.3 ± 1.3%. Hardouin et al. added six enzymes including protease and cellulase into the mixture of *Ulva* sp. and ultrapure water to react at 50 ℃ for 3 h, heated to 90 ℃, and inactivate the enzyme for 15 min. The yield of polysaccharide was 35.3 ± 0.3%. It is obvious that the temperature and pH value required for the reaction can be greatly reduced by using the high efficiency and mildness of the enzyme, making the reaction environment milder. In addition, the yield of the enzyme-assisted extraction method is higher than that of the solution extraction method, which can make full use of marine biomass resources, and it is a method that can effectively use marine green biomass resources such as ulvan.

To sum up, the main extraction methods of ulvan are based on hot water extraction, and various extraction methods are derived by adding different auxiliary technologies at different stages, but there are some advantages and disadvantages of each method. Yaich et al. tried to combine the enzyme-assisted extraction method with the solution extraction method and compared them with the solution extraction method. The results showed that the yield of ulvan increased from 13.06 to 17.14% by the organic combination of the two methods. In likewise, Chen et al. combined ultrasonic-assisted extraction with enzyme-assisted extraction, and compared it with hot water extraction, enzyme-assisted extraction and ultrasonic-assisted extraction, respectively. The results showed that the yield of ultrasonic-assisted extraction and enzyme-assisted extraction was 26.7 ± 0.9% higher than that of the other three extraction methods. Therefore, by organically combining different extraction methods, the yield of ulvan can be improved and the extraction cost reduced, thus promoting follow-up research on ulvan.

### Purification methods of ulvan

The crude ulvan obtained by hot water extraction and other methods contains non-polysaccharide impurities such as protein and other small molecules. It needs to be further purified to obtain high-purity polysaccharide samples that can be used for structure and activity studies. The isolation and purification methods of ulvan mainly include dialysis, ultrafiltration, and column chromatography, of which the latter is divided into ion exchange column chromatography and gel column chromatography.

To achieve the purpose of purification, dialysis primarily employs the selective permeability of membranes to remove salt and low-molecular-weight impurities from the crude extract of ulvan via a dialysis bag with appropriate molecular weight retention (Wang et al. 2016). Similarly, ultrafiltration also uses the same principle to separate the salt and small molecular solute in the crude extract through the membrane, and it also has many advantages including low cost, high efficiency, environmental protection, and continuous operation (Zhang and Wang 2016). The ultrafiltration dialysis tube and membrane filter have various filtering pore diameters or molecular weight cutoffs (MWCO). For dialysis, the selection of pore size is based on the molecular size of ulvan. However, the selection of pore size for ultrafiltration is not only based on the molecular size of ulvan but also depends on the elution amount (which decreasing with the pore size). In order to balance the influence of elution amount and pore size, the MWCO of about 10 kDa was generally selected (Glasson et al. 2022, 2017).

Nevertheless, many extracts of ulvan contain more impurities, and it is difficult to further refine and classify them only through dialysis and ultrafiltration. As ulvan is a water-soluble anionic polysaccharide, it is suitable for further purification by ion exchange chromatography (IEC) or gel filtration chromatography (GFC). As shown in Table 2, although the separation columns are quite different, they are gradient eluted and purified with NaCl as the mobile phase. Among them, Glasson et al. purified ulvan from *U*. *ohnoi,* *U*. *tepida,* and *U*. *prolifera,* respectively, and the final polysaccharide yields were 1.45, 1.29, and 2.8% (Glasson et al. 2022). Li et al. gradient purified the ulvan from *U. pertusa*, collected the components eluted by 0, 0.5, and 1.0 M NaCl, and then measured the polysaccharide content by the phenol–sulfuric acid method and determined that the main polysaccharide fraction was at 1.0 M elute (Li et al. 2020a). Through chromatography, we can obtain purer ulvan, which enables us to further study the conformation, structure, activity, and structure–activity relationship of ulvan.

### Preparation of *Ulva* oligosaccharide

Degraded ulvan, also known as *Ulva* oligosaccharide, is more bioavailability and soluble. Therefore, the degradation and preparation of *Ulva* oligosaccharides have attracted more and more attention. According to the preparation of *Ulva* oligosaccharides, they can be classified into chemical degradation, physical degradation, and enzymatic degradation.

#### Preparation of *Ulva* oligosaccharide by chemical and physical degradation

The chemical degradation method is used to prepare *Ulva* oligosaccharides by destroying the glycosidic bond in ulvan with strong acidic or strong oxidizing chemical reagents. In the process of extracting ulvan by Hela Yaicha et al., they found that a large number of low-molecular-weight components existed in the alcohol precipitated products under lower pH conditions, which provided a basis for the subsequent preparation of oligosaccharides through strong acids (Yaich et al. 2013). After that, Liu et al. and Roberta Paulert et al. degraded ulvan with H_2_SO_4_, HCl, and trichloroacetic acid and prepared oligosaccharides with molecular weights less than 3000 Da and non-sulfated dimers, respectively (Liu et al. 2019; Paulert et al. 2021). In addition, ulvan can also be degraded by strong oxidants. For example, Zhang et al. and Joel T. Kidgella et al. used H_2_O_2_ to degrade ulvan into oligosaccharide with 10.6 kDa and 6.8 kDa molecular weights, respectively (Kidgell et al. 2020; Zhang et al. 2008). Wu et al. combined H_2_O_2_ with ascorbic acid to degrade ulvan, and the molecular weight of its oligosaccharide is 956.71 Da, which is lower than before (Wu et al. 2020).

However, there are few studies on the physical degradation of ulvan, and only Yu et al. degrade ulvan through microwave and high pressure (Pengzhan et al. 2003). Furthermore, Simona et al. demonstrated that ulvan would self-hydrolyze in hot water solution at high temperature, and that by controlling the optimal temperature, 78.7% of the rare sugar rhamnose, glucuronic acid, and other minor degradation products could be recovered (Podolean et al. 2022). However, the physical degradation method requires a lot of energy to destroy the glycosidic bond in ulvan to prepare *Ulva* oligosaccharides, which has the same problems as the chemical method, such as harsh reaction conditions and a long degradation time (Tang et al. 2021).

#### Preparation of *Ulva* oligosaccharide by enzymatic degradation

Compared with chemical and physical methods for the degradation of ulvan, enzymatic degradation of ulvan has the advantages of mild reaction conditions, good product specificity, and environmental friendliness, which has attracted the extensive attention of researchers (Li et al. 2023; Tang et al. 2023). At present, the enzymes mainly used to degrade ulvan are PL24, PL25, PL28, and PL40 family ulvan lyases, which are the enzyme that specifically degrades ulvan. As shown in Table 3, LOR_107 from *Alteromonas* sp. LOR (Kopel et al. 2016), AsPL from *Alteromonas* sp. (AsPL) (Qin et al. 2020), PLSV from *Pseudoalteromonas* sp. strand PLSV (Qin et al. 2018), Uly1 from *Catenovulum maritimum* (Xu et al. 2021), LOR_29 from *Alteromonas* sp. LOR (Xu et al. 2021), FaUL from *Formosa agariphila* KMM 3901 (Konasani et al. 2018b), and FaPL28 from *Formosa agariphila* KMM 3901T (Reisky et al. 2018) have the highest activity between 30 and 45 ℃. And PLSV from *Pseudoalteromonas* sp. PLSV_3875 (Kopel et al. 2016), ALT3695 from *Alteromonas* sp. A321. ALT3695 (Gao et al. 2019), and NLR42 from *Nonlabens ulvanivorans* NLR42 (Nyvall Collén et al. 2011) showed the best activity at 50 ℃, while TsUly25B from *Thalassomonas* sp. LD5 reached 60 ℃ (Wang et al. 2022). The optimal pH of all ulvan lyases are between 7.5 and 9, showing high catalytic activity in a weak alkaline environment, which may be the adaptive evolution of marine bacteria to a weak alkaline seawater environment (Reisky et al. 2018). Ulaganathan studied the structure and degradation mechanism of PL24, PL25, and PL28 family ulvan lyase, respectively. Ulvan lyase primarily cleaved the *β*-(1 → 4)-glycosidic bond between _L_-rhamnose-3-sulfate (Rha3S) and _D_-glucuronic acid (GluA) or _L_-iduronic acid (IduA) via the *β*-elimination mechanism, producing two and four degrees polymerized (Dp) oligosaccharides (Ulaganathan et al. 2018a, 2018b, 2017). This is also the reason that almost all the degradation products of ulvan lyases are even oligosaccharides of DP2 and DP4. In addition, the *β*-elimination mechanism can effectively preserve the structural characteristics of rare sugars during the ulvan degradation, laying the groundwork for their high-value development. Furthermore, besides ulvan lyase, there are Cdf79930 from *Formosa agariphila* KMM 3901 (Konasani et al. 2018a), a lyase with broad spectrum activity, and P29_PDnc (Bäumgen et al. 2021), a dehydrating enzyme also from the same strain, which can participate in the degradation and further degrade the product into monosaccharide (Tables 4, 5, and 6). It provides different ideas for the enzymatic degradation of ulvan (Li et al. 2020b).

**Table 3:** Different extraction methods of ulvan

Method	Advantages	Disadvantages
Hot water extraction	A. Low cost	A. It takes a lot of time
	B. Simple and easy to operate	B. Low product yield
		C. High temperatures may cause degradation of certain polysaccharides
Acid–Base extraction	A. Save time	A. May disrupt polysaccharide structure
		B. Increased cost of waste liquid treatment
Physical-assisted extraction	A. Save time	A. May disrupt polysaccharide structure
	B. High extraction efficiency	B. Cannot improve polysaccharide yield and purity
	C. Maintain the structure and biological activity of polysaccharide	
Enzyme-assisted extraction	A. Mild reaction conditions	A. High cost
	B. High extraction efficiency

**Table 4:** The summary of methods for purification of ulvan

Method	Separation column	Mobile phase	Flow speed	Refs.
	Q Sepharose XL	0–2 M NaCl	5.0 ml/min	(Glasson et al. 2022)
IEC	Q Sepharose XL	0–2 M NaCl	20.0 ml/min	(Kidgell et al. 2020)
	DEAE-Sepharose	0–1 M NaCl	10.0 ml/min	(Li et al. 2020a)
GFC	HiTrap Q FF	0–2 M NaCl	2.0 ml/min	(Chi et al. 2021)
	HiTrap Q FF	0–2 M NaCl	2.0 ml/min	(Chi et al. 2020a)

**Table 5:** Different preparation methods of *Ulva* oligosaccharide

Method	Advantages	Disadvantages
Chemical degradation	A. Simple operation B. Inexpensive	A. Harsh reaction conditions
Physical degradation		B. Long processing time
Enzymatic degradation	A. Mild reaction conditions B. Excellent product specificity	A. Enzyme are expensive

**Table 6:** The properties of ulvan lyase from different sources

Sources	PL family	Optimal pH	Optimal temperature ( °C)	K_m_	Products	Refs.
*Alteromonas* sp. LOR	PL24	8.0	40	-	DP2, DP4	(Kopel et al. 2016)
*Alteromonas* sp. (AsPL)	PL24	8.5	40	3.19 ± 0.37 mg·mL^−1^	DP2, DP4	(Qin et al. 2020)
*Pseudoalteromonas* sp. strain PLSV	PL24	8.0	35	2.10 ± 0.31 mg·mL^−1^	DP2	(Qin et al. 2018)
*Pseudoalteromonas* sp. PLSV	PL24	8.0	50	-	DP2, DP4	(Kopel et al. 2016)
*Catenovulum maritimum*	PL24	9.0	40	-	DP2	(Xu et al. 2021)
*Alteromonas* sp. LOR	PL25	7.5	45	-	DP2	(Foran et al. 2017)
*Alteromonas* sp. A321. ALT3695	PL25	8.0	50	0.43 mg·mL^−1^	DP2, DP4	(Gao et al. 2019)
*Thalassomonas* sp. LD5	PL25	9.0	60	1.01 ± 0.05 mg·mL^−1^	DP2, DP4	(Wang et al. 2022)
*Nonlabens ulvanivorans* NLR42	PL28	9.0	50	5.1 ± 0.2 mg·mL^−1^	DP2, DP4	(Nyvall Collén et al. 2011)
*Formosa agariphila* KMM 3901	PL28	8.5	45	3.0 ± 0.1 mg·mL^−1^	DP2	(Konasani et al. 2018b)
*Formosa agariphila* KMM 3901^ T^	PL28	8.5	29.5	0.26 ± 0.06 g·L^−1^	DP2	(Reisky et al. 2018)

## Biological activity of ulvan and *Ulva* oligosaccharide

### Biological activity of ulvan

In recent years, with the continuous development of the extraction and purification technology of ulvan, more and more researchers have studied the activities of ulvan obtained from different extraction processes. At present, biological activities such as anti-virus, anti-inflammatory, antioxidant, anticoagulant, immune regulation, and induced plant defense have been reported by researchers.

#### Anti-virus activity

**Fig. 2:**
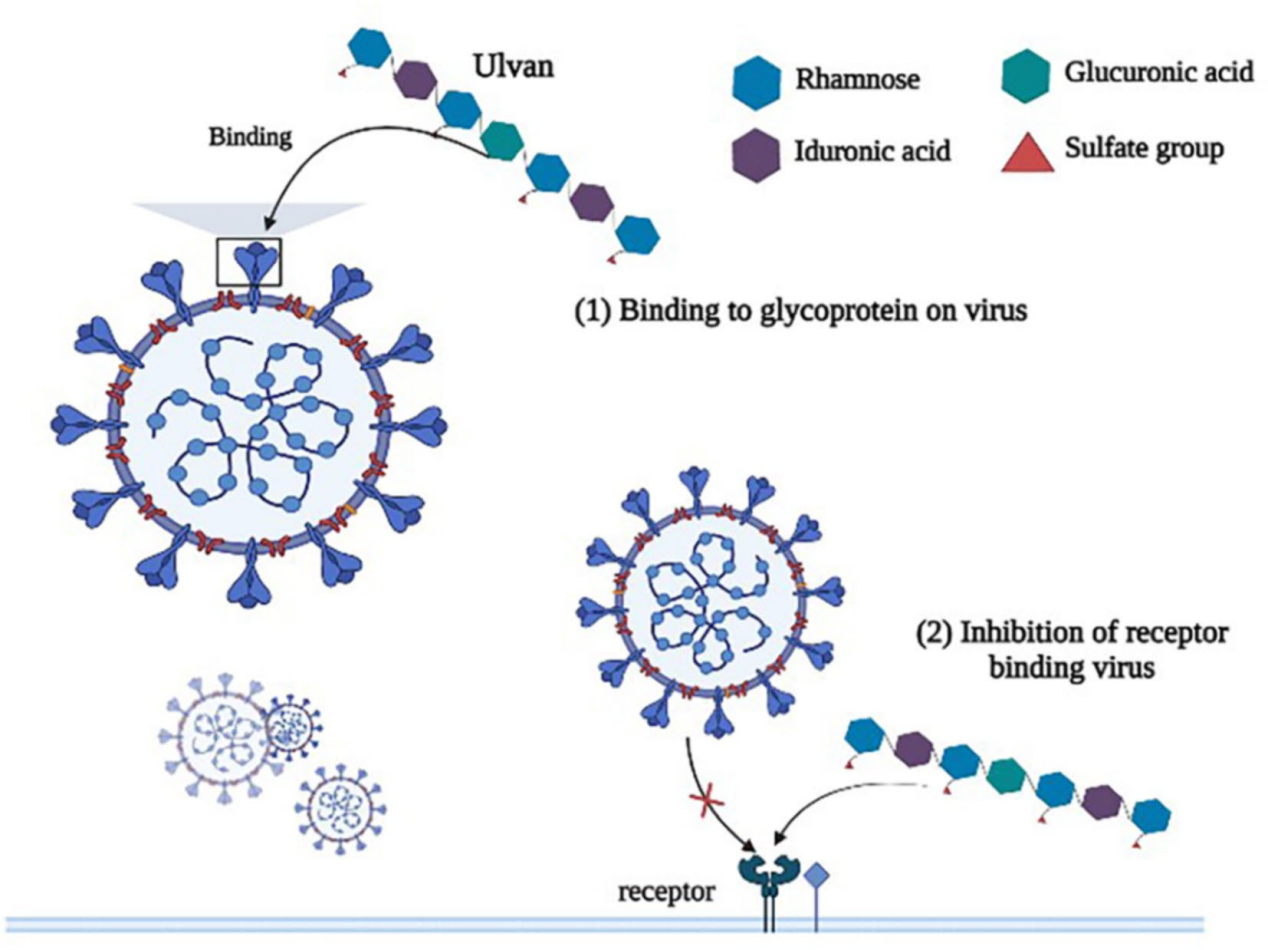
The putative antiviral mechanism of ulvan

Much research shows that ulvan from different *Ulva* sp., such as *Ulva compressa*, *U. lactuca*, *U. clathrata*, *U. intestinalis*, *U. armoricana*, and *U. pertusa*, have the activity of anti-virus (Aguilar-Briseño et al. 2015; Hardouin et al. 2016; Lopes et al. 2017; Morán-Santibañez et al. 2016; Song et al. 2016). Ulvan is a natural sulfated polysaccharide, which in turn can have unique inhibitory effects on viruses and tumors. Recently, with the increasingly serious COVID-19 epidemic, Shefer et al. evaluated the antiviral SARS CoV-2 activity of ulvan extracted by ammonium oxalate (AOx scheme) and HCl (HCl scheme), respectively. The research results showed that ulvan extracted by the AOx scheme could protect VERO E6 cells from the cytopathic effect of SARS CoV-2, and they attributed it to the interaction of negatively charged groups of ulvan with the protein on the surface of the virus (Shefer et al. 2021). Sulfated polysaccharides bind to viral binding sites (CD_4_ receptors) on the surface of T cells, interfering with viral invasion, which may be due to the binding of sulfate groups to the polysaccharides through electrostatic interactions. The research of Chi et al. on the anti-vesicular stomatitis virus activity of ulvan also has similar research results, that is, ulvan may inhibit virus infection and replication by interacting with viral envelope glycoproteins or binding with cell surface receptors. And they also found that the higher-molecular-weight ulvan (38.5 kDa) had better antiviral activity, probably because the longer glycan chains more readily recognized and interacted with proteins attached to the viral surface (Chi et al. 2020b). In fact, the putative anti-virus mechanism of sulfated polysaccharide is to bind to glycoprotein on the virus or adsorb on the cell surface receptor to prevent the virus from entering the cell interior, as shown in Fig. 2 (Andrew and Jayaraman 2021; Lu et al. 2021).

Therefore, it can be hypothesized that molecular weight and charge also affect the antiviral activity of Ulva stramonium polysaccharides. However, an obstacle to the application of sulfated polysaccharides as antiviral drugs is that they usually have anticoagulant activity and thus side effects. Current research is directed toward the synthesis of sulfated derivatives with high antiviral activity and low anticoagulant activity.

#### Antioxidant activity

Oxidative stress is defined as an imbalance of oxidant and antioxidant levels in the body induced by reactive oxygen species (ROS), which disrupts redox signal transmission and regulation and can potentially cause molecular damage. It is implicated not only in the aging process but also in the pathophysiology of numerous illnesses (Chen et al. 2022a). As anti-oxidation research has progressed, the anti-oxidative activity of ulvan produced using various extraction techniques has been identified. For instance, Sulastri Evi et al. discovered that with the increase of the concentration of ulvan extracted with HCl in the hydrogel, its ability to scavenge hydroxyl radicals was continuously enhanced (Sulastri et al. 2021). Yuan et al. extracted ulvan by microwave-assisted extraction, which had a scavenging ability of 27.6% and 68.6% for DPPH free radicals and ABTS free radicals, respectively, and good reducing activity (Yuan et al. 2018). Chen et al. examined the antioxidant activity of ulvan extracted using hot water, enzyme-assisted extraction, microwave-assisted extraction, and microwave enzyme-assisted extraction and found that the enzyme-assisted extraction had the best DPPH scavenging capacity. In addition, Li et al. discovered that the scavenging capacity of purified ulvan (FU) and purified ulvan with high sulfate group (HFU) in hot water-extracted ulvan, FU, and HFU is superior to that of Vc, and in vivo tests on mice revealed that different dosages of HFU groups significantly increased the activities of catalase (CAT), glutathione peroxidase (GSH-Px), and superoxide dismutase (SOD), while lowering MDA levels text. These results indicate that ulvan have a strong capacity to scavenge DPPH and ABTS free radicals in vitro, probably because the hydroxyl groups of the polysaccharides provide hydrogen ions, leading to the reduction of DPPH radicals. Ulvan achieved antioxidant activity via influencing the activities of CAT, GSH-Px, and SOD in vivo. However, to determine the signaling pathways involved, further research on the method by which ulvan alters the expression of antioxidant enzymes in vivo is required.

#### Anti-hyperlipidemic activity

Hyperlipidemia is a major cause of vascular disease, and traditional treatment techniques have significant negative effects. As a result, studies on anti-hyperlipidemic components in natural foods are expanding (Ge et al. 2022). Ulvan, the primary active element in the green algal *Ulva*, has long been known to have anti-hyperlipidemic properties (Pengzhan et al. 2003). Currently, all the ulvan utilized in the investigation of anti-hyperlipidemic activity is produced using hot water extraction. Sathivel et al. (2008) tested the anti-hyperlipidemic activity of crude ulvan by feeding it to mature male albino rats. The findings revealed that ulvan could significantly inhibit the acute rise in serum triglyceride, free fatty acid, and total cholesterol levels while also significantly lowering high-density lipoprotein (HDL), very low-density lipoprotein (VLDL), and having a parallel inhibitory effect on the rise in low-density lipoprotein (LDL) (Sathivel et al. 2008). Following that, Li et al. compared the anti-hyperlipidemic ability of crude ulvan (U) and purified ulvan (F1 and F2). The result shows that F1, which can dramatically lower the level of low-density lipoprotein cholesterol (LDL-C) while increasing the level of high-density lipoprotein cholesterol (HDL-C) at a dose over 250 mg/Kg, and F2, which reduced serum total cholesterol (TC) and triglycerides (TG) levels considerably, have better anti-hyperlipidemic ability than U (Li et al. 2018). Furthermore, Qi et al. and Li et al. revealed that sulfated ulvan had better anti-hyperlipidemic activity than ulvan, while the latter discovered that the anti-hyperlipidemic activity of the pure ulvan was further boosted after sulfated, that is, the LDL-C content was reduced, HDL-C was raised to normal levels, and the TC and TG contents were significantly reduced at only half the dose of FU (Li et al. 2020a; Qi et al. 2012). These findings suggest that the anti-hyperlipidemic effect of ulvan is mediated by various pathways, the balance of which is determined by the structural properties of ulvan. This opens the door to tailoring anti-hyperlipidemic supplements for more specific purposes.

#### Anticancer activity

Abd-Ellatef et al. studied the prevention of carcinogenic activity in ulvan obtained by hot water extraction on the breasts of Wistar rats. The findings revealed that ulvan could not only raise the expression of the tumor suppressor protein p53 and the enzymatic activities of glutathione-S-transferase (GST), GPx, and CAT, but also considerably lower blood TNF-α and NO (Abdellatef et al. 2017). Shao et al. investigated the inhibitory impact of ulvan produced by hot water extraction, which, after partial desulfurization, inhibited the development of DLD intestine cancer cells, and found that the large molecular weight desulfurized sample DS-UFP3 inhibited cell growth well at a concentration of 4 mg/mL (Shao et al. 2014). The anticancer activity of ulvan appears to be exerted through multiple pathways, including the encouragement of cancer cell death, the decrease of cancer cell growth, and the activation of innate immune responses. Ulvan is a class of biological response modifiers, and if it can be used as anticancer drugs, its greatest advantage is that it have fewer toxic side effects, and it can be more effective when used in combination with chemotherapeutic drugs. Moreover, to a certain extent, ulvan can counteract the adverse reactions caused by chemotherapeutic drugs.

#### Immunomodulation activity

Klongklaewad et al. investigated the immunomodulation activity of ulvan prepared by hot water extraction on Pacific white shrimp and discovered that ulvan promoted the expression of immune-related genes (Anti-lipopolysaccharide factor (ALF), prophenoloxidase (proPO), SOD, transglutaminase (Trans), lysozyme (Lyso), C-type lectin (Clectin), and lipopolysaccharide and *β*-1,3-glucan binding protein (LGBP)) to varying degrees and had a good preventive effect on yellow head virus (YHV), significantly reducing its mortality (Klongklaew et al. 2021). Fernández-Dazde et al. evaluated the immunomodulation activity of ulvan isolated from sodium oxalate and discovered that ulvan can increase reactive oxygen species in macrophages (Fernández-Díaz et al. 2017). Ulvan is an immunostimulant that significantly promotes phagocytosis by macrophages and increases the weight of the spleen, an immune organ. According to Harikrishnan et al., who examined the immunomodulation activity of ulvan on Labeo rohita, it was discovered that ulvan can significantly increase phagocytic (PC) activity, respiratory burst (RB), alternative complement activity (ACP/ACH_50_), lysozyme (Lyz) activity, immunoglobulin M (IgM), and other cytokines or protein mRNA expression (Harikrishnan et al. 2021). In addition, ulvan promotes the expression of antioxidant-related genes (SOD, GPx, natural killer cell enhancer factor *β* (NKEF-*β*) gene, etc.) and anti-inflammatory-related genes (toll-like receptor 22 (TLR22), interleukin 1*β* (IL-1*β*), tumor necrosis factor α (TNF-α), etc.). Ulvan can achieve immunomodulatory effects by promoting the expression of immune-related genes and elevating the activities of PC, RB, and ACP/ACH_50_. In summary, it can be hypothesized that ulvan can play an antiviral role by regulating the body's immune status, enhancing the body's immunity, and promoting the value-added of T and B lymphocytes.

#### Plant defense activity

Ulvan also has positive impacts on plant defense. For example, the induction activity of thermochemically extracted ulvan on the defensive response system of table grapes was assessed by Shomron et al. The outcomes demonstrated that ulvan derived from *Ulva rigida* may raise active oxygen levels and catalase, superoxide dismutase, and chitinase enzyme activities, decreasing table grape rot (Shomron et al. 2022). According to Borba et al.'s study on wheat-to-wheat yeast resistance induced by ulvan extracted by hot water, while ulvan cannot directly play an antifungal function, it can indirectly activate the genes expressing PR proteins (PR-2 and PR-3), ROS metabolism (OXO), and the octadecanoid-based pathway (LOX and AOS). However, the utilization of ulvan would face challenges, including poor bioavailability and solubility brought on by its high molecular weight (de Borba et al. 2021). Paulert et al. investigated the immunomodulatory activity of hot water-extracted ulvan in parsley and basil, finding that ulvan can increase the levels of salicylic acid (SA), salicylic acid *β*-glucoside (SAG), and abscisic acid (ABA) in parsley and basil, as well as increase jasmonic acid (JA) accumulation in basil [86]. Ulvan demonstrated considerable bioinducer activity as well as the ability to operate as a promoter, improving plant health and resistance.

**Fig. 3:**
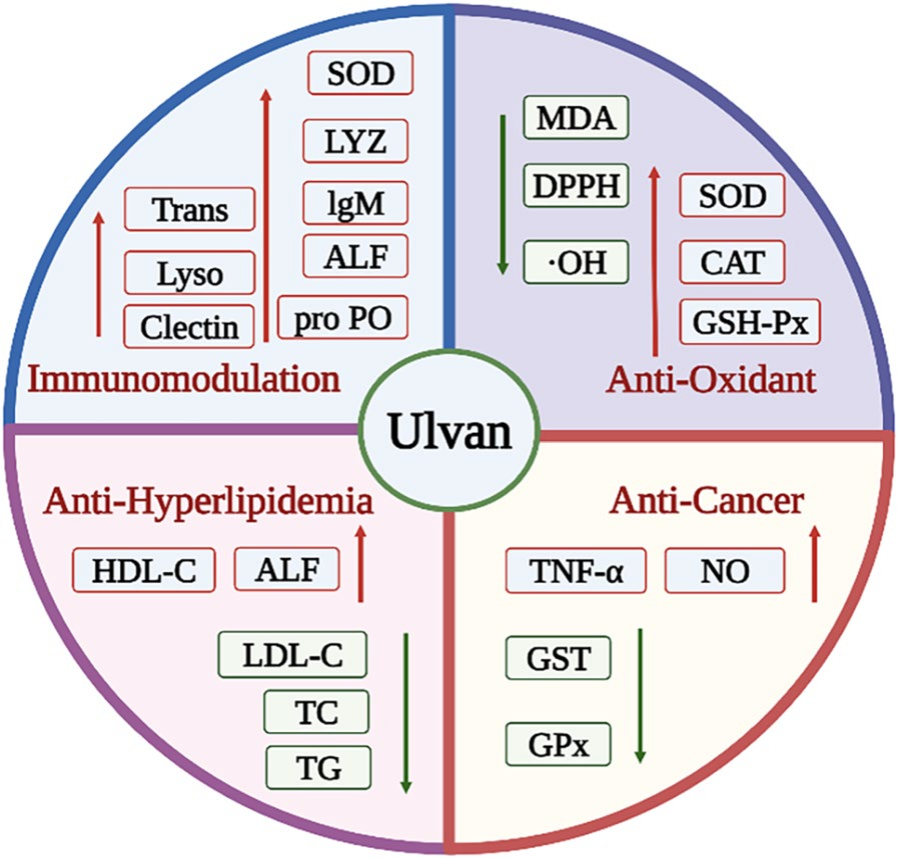
The mechanisms related to the biological properties of ulvan

In conclusion, the biological activities of ulvan, such as anti-virus, anti-oxidation, anti-hyperlipidemic, anticancer, and immunomodulation, are strongly connected to its fundamental biological activities, and they have some regulatory effects on plant defense systems. It is important to note that the biological activity of ulvan is highly connected to their molecular weight, structural properties, and content. The mechanism related to the biological activity of ulvan is shown in Fig. 3.

### Biological activity of *Ulva* oligosaccharide

**Fig. 4:**
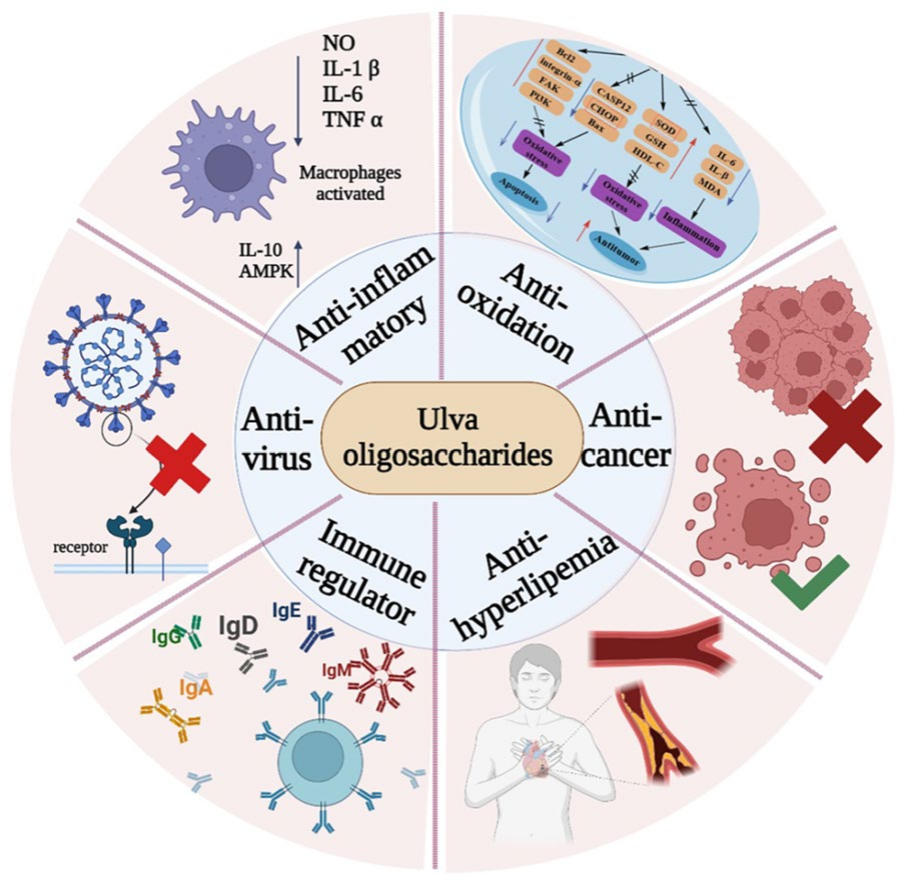
The biological activities of *Ulva* oligosaccharides

At present, the relevant research and reports on the activity of *Ulva* oligosaccharides are increasing, but the literature reports are relatively scattered, and due to the complexity of the structure of *Ulva* oligosaccharides, the mechanism of related activities and the structure–activity relationship of oligosaccharides have not yet been determined. According to Chi et al.'s evaluation of the anti-vesicular stomatitis virus (VSV) activity of *Ulva* oligosaccharides made by ulvan lyase of the PL25 family, lower-molecular-weight degraded *Ulva* oligosaccharides demonstrated antiviral activity equal to that of undegraded ulvan at 100 L/mL. Low-molecular-weight polysaccharides are preferable for the creation of dietary supplements and medications due to their comparable action to natural polysaccharides (Chi et al. 2020b). Using an enzymatic method, *Ulva* oligosaccharides were prepared by Li et al. to study their anti-inflammatory bowel disease (IBD) properties. The study's findings revealed that *Ulva* oligosaccharides started to have a protective effect on IBD at a dose of 50 mg/kg and were most effective at 100–120 mg/kg. *Ulva* oligosaccharides may also lessen the harm dextran sodium sulfate (DSS) causes to colonic epithelial cells (Li et al. 2020c). According to Tabarsa et al., *Ulva* oligosaccharides with a high sulfate group composition and a low molecular weight may successfully multiply RAW264.7 macrophages, proving that they have a higher bioavailability. Additionally, *Ulva* oligosaccharides may stimulate RAW 264.7 cell production of cytokines with weak immunomodulatory activity, such as nitric oxide, IL-1*β*, TNF-*α*, IL-6, IL-10, and IL-12 (Tabarsa et al. 2018). And Berria et al. also found that the mRNA and protein expression of cytokines (such as CCL20, IL-8, and TNF-α) were increased when porcine intestinal epithelial (IPEC-1) cells were treated with *Ulva* oligosaccharides (Berri et al. 2017). The presence of *Ulva* oligosaccharides that have been degraded might enhance the anticancer activity of ulvan, according to research by Carvalho et al. on the cytotoxicity of *Ulva* oligosaccharides on human cervical cancer cells (de Carvalho et al. 2020). In contrast to high-molecular-weight ulvan, degraded *Ulva* oligosaccharides displayed stronger antioxidant activity, according to Qi et al. (2005). Using a male Wistar rat model, Yu et al. investigated the anti-hyperlipidemic activity of *Ulva* oligosaccharides and discovered that it increased HDL cholesterol by 2.0 times and decreased TG by 46.4% in rats given *Ulva* oligosaccharide. Accordingly, degraded *Ulva* oligosaccharides were superior to undegraded ulvan in treating hyperlipidemia caused by diabetes. The biological activities related to *Ulva* oligosaccharides are shown in Fig. 4.

In terms of enhancing plant defense activity, *Ulva* oligosaccharides also performed well. Paulert et al. investigated the oxidative burst activity of *Ulva* oligosaccharides prepared by acid hydrolysis to induce dicotyledonous plants, and the findings revealed that low-molecular-weight *Ulva* oligosaccharides can have the same activity as ulvan, and its inducing activity is not dependent on acid sulfation. Because of their solubility or high viscosity, large molecular weight polysaccharides are challenging to utilize in normal agricultural contexts (Paulert et al. 2021). AbourachaaZ et al. investigated the effects of ulvan and ulvan oligosaccharides produced by ulvan lyase on apple defense reactions and corruption. *Ulva* oligosaccharides may totally prevent the formation of blue and cyan molds on fruits, according to the findings. *Ulva* oligosaccharides, as opposed to ulvan, can also better stimulate the immune regulation system of apples, resulting in the activation of antioxidant-related enzymes and an increase in the activities of phenylalanine ammonia lyase (PAL), peroxidase (POD), and polyphenol oxidase (PPO) (Abouraïcha et al. 2015). However, researches on the action of Ulva oligosaccharides are limited. This is also due to the complexity of the structure of ulvan, and there is no suitable method for obtaining oligosaccharides with a specified fine structure to research the structure–activity connection of oligosaccharides.

## Conclusion and outlook

The extraction rate of ulvan increased significantly as the extraction process was continuously optimized, going from a low yield of 7%–24% (Chen et al. 2021; Paradossi et al. 1999; Toskas et al. 2011) in the traditional extraction method to 35.3 ± 0.3% (Hardouin et al. 2016) in the enzyme-assisted extraction method and 40.4 ± 3.2% (Magnusson et al. 2019) in the microwave-assisted extraction method. In addition, obtaining high-purity ulvan by purification played a key role in the study of their structure and activity. As Glasson analyzed the monosaccharide composition and structure by purifying the polysaccharides from different sources and analyzed the in vivo and in vitro antioxidant activity and enzyme inhibitory activity of purified ulvan with a higher confidence level in their results. Enzymatic preparation of *Ulva* oligosaccharides has milder reaction conditions and higher reaction efficiencies and produces oligosaccharides with unsaturated bonds at the non-reducing end compared to chemical and physical methods. In addition, the rich biological activities of ulvan and *Ulva* oligosaccharides make them potentially applicable in food, medicine, cosmetics, and other fields.

However, studies on the structure and biological properties of ulvan are still at an early stage compared to other marine sulfate polysaccharides, carrageenan, and fucoidan. On one hand, the high-value development of ulvan is still affected by the immaturity and low purity of the large-scale preparation process, i.e., the industrial production of high-purity ulvan cannot be realized. On the other hand, the relationship between the physicochemical properties of ulvan and their biological activities, as well as the mechanism of action of the biological activities, remains unclear. This is due to the fact that studies related to the highly refined structure–function properties as well as the activity of fully characterized ulvan against highly precise targets are still scarce. In contrast, *Ulva* oligosaccharides, as degradation products of ulvan, retain various physiological activities and other excellent properties of ulvan. Although the tool enzyme ulvan lyase has been identified and certain research findings have been obtained, the reported enzymes still cannot match the demands of commercial applications due to a lack of sequence information, low stability, and low activity. As a result, ulvan lyases with high activity and exceptional stability, as well as their entire sequence information, are urgently required for commercial applications. Furthermore, precise fine structure analysis of ulvan and its oligosaccharides plays a significant role in increasing the structure–activity connection of ulvan and its oligosaccharides, as well as the high-value development and effective exploitation of ulvan. Ulvan, a rich green marine biomass resource, will be more fully utilized in the future with the realization of industrialized production of high-purity ulvan, the discovery of more and more excellent biochemical properties of ulvan lyases, and the clarification of the structure–activity relationship between ulvan and its oligosaccharides.

## Data Availability

Data may be made available on request.
